# Cone-Beam Computed Tomography Evaluation of Bone Density After Sagittal Split Osteotomy Using the Novel Modification of Low Z Plasty Technique

**DOI:** 10.3390/medicina62010062

**Published:** 2025-12-28

**Authors:** Passorn Nuntapolchai, Siripatra Patchanee, Chanekrid Oupadissakoon, Phetcharat Chatmongkhonkit, Narissaporn Chaiprakit

**Affiliations:** 1 Division of Oral and Maxillofacial Surgery, Faculty of Dentistry, Thammasat University, Pathum Thani 12120, Thailand; nuntapolchai@gmail.com (P.N.);; 2 Division of Orthodontics, Faculty of Dentistry, Thammasat University, Pathum Thani 12120, Thailand; siripatra.p@hotmail.com; 3 Thammasat University Research Unit in Mineralized Tissue Reconstruction, Thammasat University, Pathum Thani 12120, Thailand

**Keywords:** bilateral sagittal split osteotomy, novel modification of low Z plasty, bone healing, cone-beam computed tomography

## Abstract

*Background and Objectives*: This study aimed to assess the pattern and quantity of bone regeneration after mandibular setback surgery using a novel modification of the low Z plasty (NM-Low Z plasty) technique by measuring bone density (Hounsfield unit) at the osteotomy site over a 12-month postoperative period using cone-beam computed tomography (CBCT). *Materials and Methods*: This retrospective cohort study included six patients with skeletal Class III deformity who underwent bilateral sagittal split osteotomy (BSSO) setback using the NM-Low Z plasty technique between 2021 and 2023 at Thammasat University Hospital. CBCT images were obtained preoperatively and at 1 month, 6 months, and 12 months postoperatively. Bone density at the buccal, cancellous, and lingual aspects of the osteotomy gap was measured using Blue Sky Plan 4 software. The intraclass correlation coefficient was used to determine reliability. Descriptive statistics, repeated-measures analysis of variance and multiple linear regression analysis were performed for comparisons. *Results*: At 12 months postoperatively, bone density in all measured regions was not significantly different compared to the postoperative measurements, indicating sufficient bone regeneration. The cancellous and lingual cortical regions exhibited earlier recovery than the buccal cortex. No postoperative complications such as wound infection, delayed union, or non-union were reported. *Conclusions*: BSSO using the NM-Low Z plasty technique offers reliable bone healing outcomes with stable bone regeneration, thereby providing a viable alternative to conventional BSSO techniques. Radiographic evidence confirmed its clinical applicability and potential to reduce the incidence of postoperative complications.

## 1. Introduction

Bone density evaluation after bilateral sagittal split osteotomy (BSSO) helps medical practitioners understand bone regeneration timelines to directly inform clinical decisions regarding the safe timing for miniplate and screw removal, thereby reducing risks to patients and improving long-term outcomes. Mandibular prognathism and retrognathism are caused by overgrowth and undergrowth of the mandible, respectively. They affect not only the facial appearance but also function and quality of life. Orthognathic surgery plays a pivotal role in correcting dentofacial deformities that cannot be managed through orthodontic treatment alone. Bilateral sagittal split osteotomy (BSSO) remains the cornerstone surgical technique for mandibular repositioning in the management of dentofacial deformities, particularly mandibular prognathism [1]. Intraoperatively, the mandible is divided along three cutting lines (horizontal cut, sagittal cut, and vertical cut) into two parts (distal segment and proximal segment). After aligning the jaw in the proper position, plates and screws are used to secure the two segments [2]. In 2016, Tangarturonrasme et al. introduced a modified BSSO technique called the low Z plasty technique, to improve outcomes and reduce complications. The low Z plasty technique is advantageous because it allows simultaneous correction of open bite and mandibular prognathism. Moreover, it shifts the rotation area from between the posterior border of the mandible and the lingula to the retromolar region, resulting in minimal muscle interference [3]. Over the past decades, several modifications of the conventional BSSO technique have been proposed to reduce surgical morbidity, improve biomechanical stability, and optimize healing conditions. Among these, the novel modification of the low Z plasty (NM-Low Z plasty) technique has demonstrated promising biomechanical advantages, including reduced stress on fixation devices, improved load distribution, and enhanced segmental stability as evidenced by finite element analyses. In NM-Low Z, the horizontal cutting line is positioned above the retromolar area, but below the cutting line used in the Hunsuck and Epker technique (HE). NM-Low Z is less invasive to the oral soft tissue and requires less operating time. Additionally, this technique allows larger mandibular setbacks and a higher degree of rotation [4], making it highly effective for correcting severe skeletal discrepancies such as prognathism and anterior open bite. Furthermore, it improves patient safety and stability by minimizing the risk of vital structure interruption and imparting less torque to the mandibular condyle. The Finite Element (FE) analysis provides strong quantitative evidence supporting these clinical benefits. The NM-Low Z model consistently demonstrated a lower maximal equivalent von Mises (EQV) stress on the fixation materials, notably showing approximately half the stress of the HE models at 1-week post-operation. This reduced stress indicates a lower risk of fixation failure and screw loosening. The improved mechanical environment is further corroborated by the lower bone stress around the screws and lower relative displacement between the bone segments when compared to the HE technique. Crucially, the elastic strain values at the fracture site for both techniques were confirmed to be within the optimal range for initial bone healing. In conclusion, the NM-Low Z technique is not merely comparable to the conventional HE technique but offers biomechanical performance that facilitates postoperative skeletal stability by actively reducing mechanical loads on fixation materials. Its combined advantages of increased surgical range, reduced invasiveness, and superior stress distribution position it as an acceptable and potentially preferable technique for mandibular setback procedures in orthognathic surgery [5,6].

Despite the widespread clinical adoption and numerous technical modifications of BSSO, postoperative bone healing at the osteotomy site continues to be a critical determinant of long-term skeletal stability, fixation integrity, and the safe timing of hardware removal. Inadequate or delayed bone regeneration may increase the risk of complications such as fixation failure, relapse, or delayed union, thereby directly influencing patient outcomes and postoperative management strategies. The previous reports of NM-Low Z plasty mechanical performance have been well characterized, but clinical and radiographic evidence regarding bone regeneration following NM-Low Z plasty remains scarce.

Radiographic evaluation of bone healing is essential for objective postoperative assessment [7]. Cone-beam computed tomography (CBCT) has emerged as a valuable imaging modality, offering three-dimensional visualization of osseous architecture with lower radiation exposure compared to conventional computed tomography. Moreover, CBCT-derived radiographic bone density measurements, expressed in Hounsfield units (HU), provide a non-invasive means to longitudinally monitor bone regeneration and remodeling at the osteotomy site. Nevertheless, longitudinal CBCT-based assessments of bone density following BSSO using the NM-Low Z plasty technique have not yet been systematically reported.

Therefore, this study aimed to determine whether bone density returns to the pre-treatment level within 12 months after BSSO using the NM-Low Z plasty technique. Specifically, we aimed to compare postoperative bone density at three different postoperative timepoints with the preoperative density using HU derived from CBCT images. Additionally, we aimed to evaluate whether the NM-Low Z technique influences healing rates.

The findings in this study provide insights into the biological healing outcomes after NM-Low Z plasty and support the use of CBCT as a clinical tool for postoperative monitoring in orthognathic surgery. Understanding the density of regenerated bone can help guide clinical decisions, such as the appropriate timing for the removal of miniplates and screws.

## 2. Methods

### Study Design and Patients

This retrospective study included patients with skeletal Class III deformity, who underwent BSSO using the NM-low Z technique at Thammasat Hospital between 2021 and 2023.

The inclusion criteria consisted of the following:

(1) Patients aged 18–40 years old;
(2) Patients who were diagnosed with mandibular prognathism;
(3) Patients who underwent mandibular sagittal split osteotomy (setback) by NM-Low Z technique from 2021 to 2023 at Thammasat University Hospital. All osteotomy sites were stabilized using titanium miniplates and screws in a standard fixation technique.
(4) No bone grafts, bone substitutes, or platelet concentrates (e.g., PRP or PRF) were used in the osteotomy gaps for any patient, ensuring that the observed bone regeneration was solely due to the natural biological healing process supported by the NM-Low Z plasty technique and rigid fixation.

The exclusion criteria consisted of the following:

(1) Patients who underwent genioplasty;
(2) Pregnancy within the time of follow-up (1 year after surgery);
(3) Patients with the following diseases associated with bone metabolism: osteoporosis, rickets, osteomalacia, scurvy disease, hyperparathyroidism, hypoparathyroidism, hyperpituitarism, or hypopituitarism;
(4) Intake of the following drugs or dietary supplements related to bone mineralization: calcium, phosphate, or magnesium;
(5) Having a favorable or unfavorable fracture line during surgery, which was recorded in the operative note;
(6) Smoking within 4 weeks before surgery or during the time of follow-up.
(7) Patients who underwent chemotherapy or radiotherapy in the head and neck area.

Note: Standardized postoperative care included liquid diet restrictions for 4 weeks, prophylactic antibiotics, and pain management. Intermaxillary fixation (IMF) was used for 2 weeks postoperatively.

**Figure 1:**
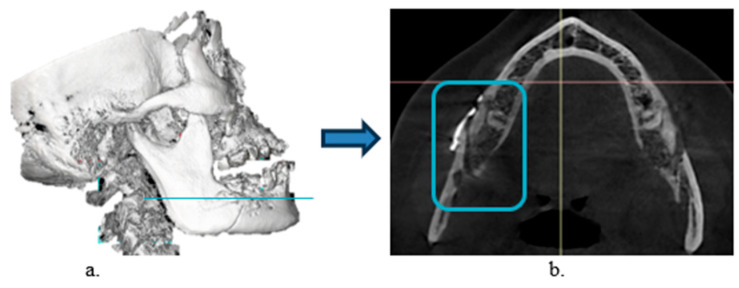
Cross sectional area of interest. (**a**) Location of the junction between the head and body of the first screw in the proximal segment (blue line). (**b**) Axial DICOM image; rectangle indicates area of interest.

**Figure 2:**
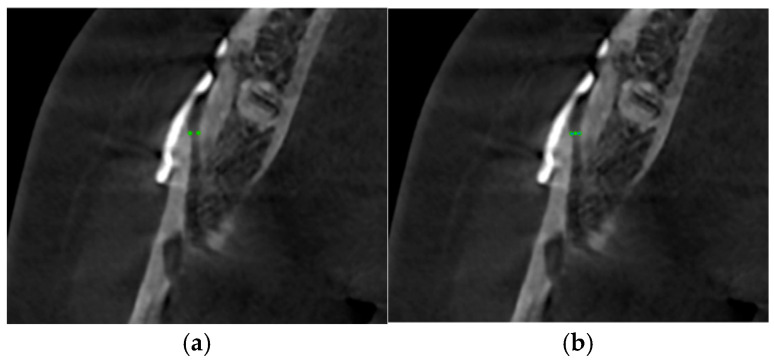
Measurement of buccal bone density. (**a**) Points indicate bone density measurement location at the buccal cortex. (**b**) Points indicate bone density measurement location at the buccal cortex point and midpoint of the line joining the two buccal points. Line indicates gap width along the X axis between the two buccal points.

**Figure 3:**
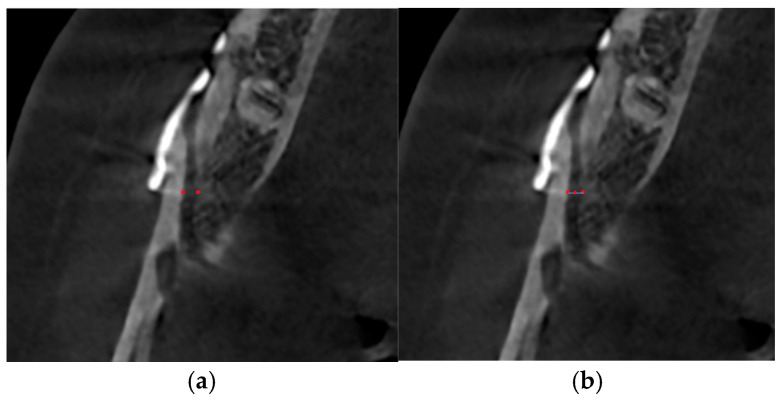
Measurement of cancellous bone density. (**a**) Points indicate location of cancellous bone density measurement. (**b**) Points indicate location of cancellous bone density measurement. Line indicates gap width along the X axis between two cancellous points.

**Figure 4:**
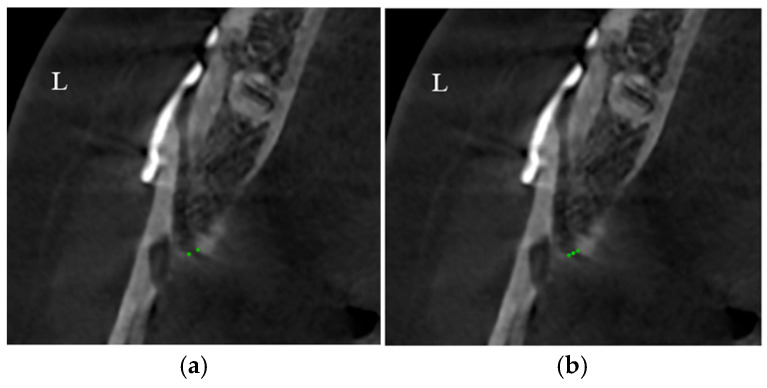
Measurement of lingual bone density. (**a**) Points indicate location of bone density measurement at the lingual cortex. (**b**) Points indicate location of bone density measurement at the lingual cortex point and midpoint of the line joining two lingual points. Line indicates gap width along the X axis between the two lingual points.

**Figure 5:**
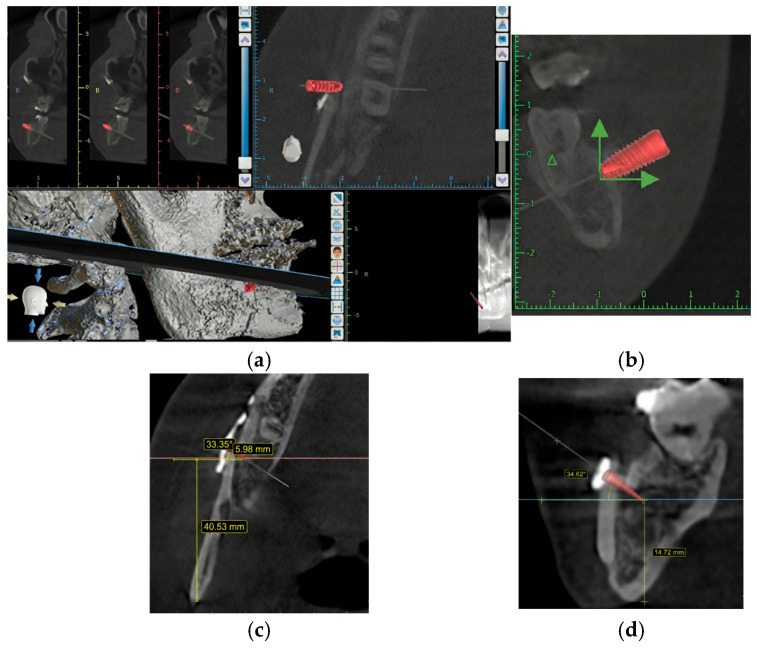
Determination of the implant axis and location. (**a**) Implant fixture inserted using Blue Sky Plan 4 software. (**b**) Green arrows were constructed to indicate angulation of a dental implant along the X (horizontal) and Y (vertical) axis. (**c**) Measurement of distance from the posterior border of the ramus and angulation in the sagittal plane. (**d**) Measurement of distance from the inferior border of the mandible and angulation in the sagittal plane.

**Figure 6:**
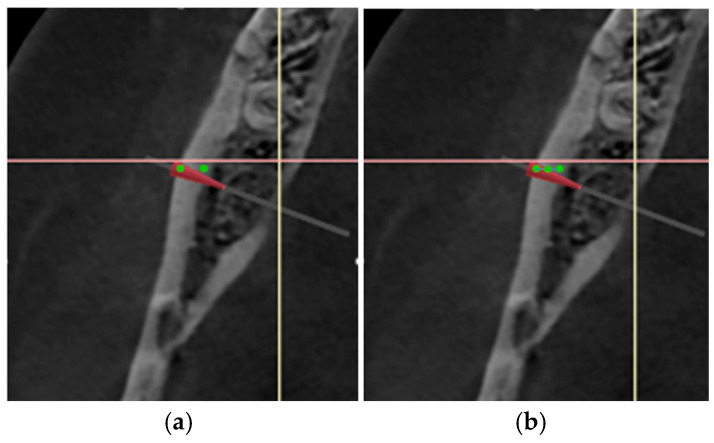
Axial image at T2 showing area of interest. (**a**) Points indicate location of buccal bone density measurement at T2. (**b**) Points indicate location of bone density measurement at the buccal cortex point and midpoint of the line joining the two buccal points. Blue line indicates gap width along the X axis between two buccal points.

We calculated the effect size following the procedure from Ueki. et al., 2015 [8], who discovered changes in the computed tomography (pixel) value of the mandibular ramus bone and fixation screw after sagittal split ramus osteotomy using the G-power version 3.1; the effect size is 1.86, the power is 0.95, and the result includes data from six samples. The procedure was as follows:

(1) CBCT (Sirona Dental System GmbH, Bensheim, Germany) was performed at four timepoints using the following imaging parameters: scanning time, 16.5 s; voltage, 85 kV; current, 6 mA; exposure, 1075 mGycm^2^; field of view, 17 cm; slice thickness, 0.25 mm; voxel size, 800 × 896 × 792.
(2) CBCT data were exported from Sidexis 4 dental imaging software (Version 4.3.1.0 Revision 70140; Dentsply Sirona, Bensheim, Germany) into a Digital Imaging and Communications in Medicine (DICOM) file.
(3) The DICOM file was imported into Blue Sky Plan 4 treatment-planning software (Version 4.9.4; Libertyville, IL, USA).
(4) CBCT images were reoriented with the inferior border of the proximal segment parallel to the floor (Figure 1a).
(5) Axial CBCT images were selected because the sagittal cut of BSSO is the most obvious in the axial view (Figure 1b)
(6) The section showing the junction between the head and body of the first screw in the proximal segment adjacent to the vertical cut was selected because previous studies have shown that this point is a stress-bearing area [6].

(7) Buccal point: Bone density (HU) in the gap created by the sagittal cut adjacent to the margin was measured at the level of the first screw (Figure 2, green points). The gap width along the X axis between two buccal points and bone density (HU) at the midpoint of the line joining the two buccal points was measured.

(8) Cancellous popoint: The bone density (HU) in the gap created by the sagittal cut was measured adjacent to the margin at the level of the most posterior part of the titanium plate (Figure 3a, red points). The gap width along the X axis between two cancellous points and bone density (HU) at the midpoint of the line joining two cancellous points were measured.

(9) Lingual point: The bone density (HU) in the gap created by the sagittal cut was measured adjacent to the margin at the midpoint of the lingual cortex (Figure 4a, green point). Gap width along the X axis between two lingual points and bone density (HU) at the midpoint of the line joining two lingual points were measured.

(10) The distances from the buccal point to the cancellous and lingual points along the X and Y axes and from the buccal point to the most buccal margin were recorded.
(11) Reference registration: In the preoperative (T0) radiograph, the screw was substituted with an implant fixture (size, 2 × 7 mm) in the Blue Sky Plan 4 software, and its position (distance from inferior border of the mandible and posterior border of the ramus) and angulation along the X and Y axes in the axial, sagittal, and coronal planes were recorded (Figure 5).

(12) Bone density (HU) was measured at the distance from the buccal margin that was measured in 11 on the preoperative radiograph (Figure 6, green points).

(13) All measurements were repeated after 2 weeks to analyze the reliability of the test.
(14) Steps 1–13 were repeated for other radiographs (24 CBCT images from 6 patients)
(15) Data collection and statistical analysis were performed using IBM SPSS Statistics software version 29.0.

## 3. Results

A total of six patients (four men and two women), aged 23–36 years, underwent BSSO using the NM-Low Z plasty technique during the study period. The average mandibular setback distance was 11.87 mm, and the osteotomy gap ranged from 0.78 mm to 1.88 mm (range of gap healing) (Table 1).

**Figure 7:**
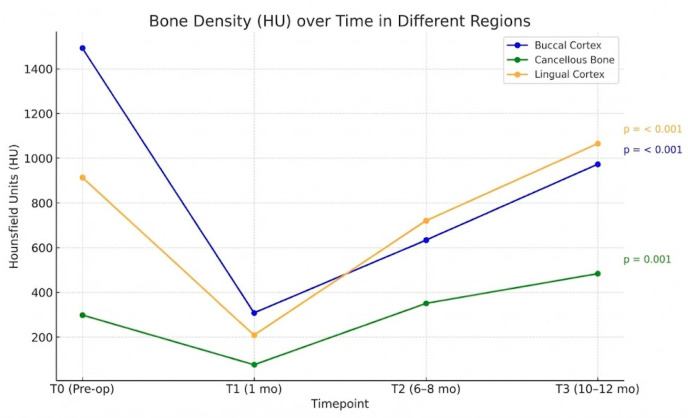
Line graphs showing the trend of bone density over time in different areas. No cases of postoperative wound infection, delayed union, or non-union were observed.

Bone regeneration was evaluated by comparing bone density (HU obtained from CBCT) at three postoperative time points—<1 month (T1), 6–8 months (T2), and 10–12 months (T3)—with that at T0 (Figure 7). The intraclass correlation coefficient for intraobserver reliability was >0.90 for all measured points, indicating excellent measurement consistency (Table 2).

Bone density of the buccal cortex, cancellous bone, and lingual cortex was measured. The Shapiro–Wilk test indicated that the data for buccal cortical bone density (*W* = 0.934, *p* = 0.120), cancellous bone density (*W* = 0.951, *p* = 0.291), and lingual cortical bone density (*W* = 0.941, *p* = 0.169) were normally distributed (Table 3).

**Figure 8:**
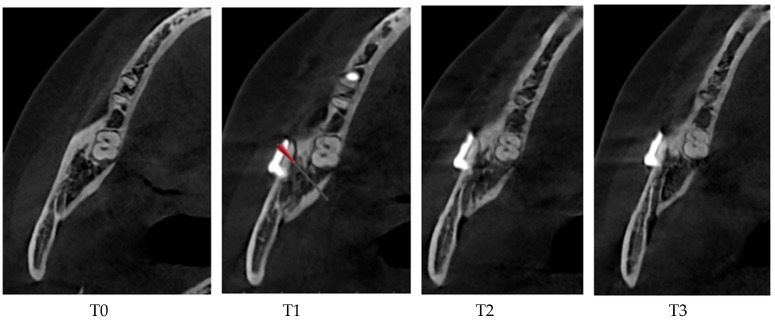
Cross-sectional view of the osteotomy site showing bone density change from baseline to the different healing phases.

Statistical analysis using repeated-measures analysis of variance revealed the following. At T1, the buccal and lingual cortex areas exhibited a significant decrease in HU values. Moreover, HU values of the cancellous bone at T1, which corresponded with the initial phase of soft callus formation, were not significantly different from those at T0. At T2, the cancellous and lingual regions showed marked improvement in bone density, with HU values approaching or exceeding those at T0. The buccal cortex demonstrated more gradual healing, reaching a comparable density to baseline at T3 (Table 4) (Figure 8).

Table 5 summarizes the influence of two independent variables, setback distance and mean gap size, on the dependent variable, bone density at 10–12 months (HU at T3), for each measured region.

The primary finding across the three anatomical regions (buccal, cancellous, and lingual cortex) is the lack of statistical significance for both setback distance and mean gap size in predicting bone density (HU) at T3 (10–12 months). The *p*-values for all individual predictors in the buccal and cancellous models exceeded the 0.05 threshold (ranging from 0.702 to 0.926). This suggests that we cannot confidently reject the null hypothesis that these two surgical parameters had no linear effect on bone healing in these regions. Consequently, the results should be interpreted as reflecting trends and explanatory power rather than definitive proof of causality.

Buccal cortex:

- R^2^ = 0.083: This model has the lowest explanatory power, indicating that only 8.3% of the variability in the buccal cortex’s final density is explained by setback and mean gap size. The remaining 91.7% must be attributed to biological factors (e.g., periosteal stripping and vascular compromise) or individual patient variations.
- Coefficient trend: Both setbacks (Coef. +2.20) and mean gap (Coef. +43.27) showed a positive, though non-significant, association with HU. The positive coefficient for mean gaps was paradoxical and may be a spurious result due to multicollinearity, or it might highlight the overwhelming importance of fixation stability over gap size in this slow-healing cortical area.

Cancellous bone:

- R^2^ = 0.020: this is the weakest model, explaining only 2.0% of the variability. This is consistent with the literature, which suggests that cancellous bone healing is rapid and primarily driven by local vascularity and initial stability rather than surgical parameters that only slightly vary within an acceptable clinical range.
- Coefficient trend: the mean gap coefficient was negative (−175.85). While non-significant, this negative trend is biologically sound: a wider cancellous gap requires more tissue to bridge the defect, potentially leading to lower overall density at T3 compared to an immediate repair.

Lingual cortex

The lingual cortex model provides the most compelling insights, despite the *p*-values being non-significant R^2^ = 0.774. This high value is the most critical finding. It means that 77.4% of the observed variation in final lingual bone density is collectively explained by setback distance and mean gap size. This suggests that the surgical management of the osteotomy site is the dominant factor determining the quality of bone regeneration in the lingual area, unlike the buccal and cancellous regions.

## 4. Discussion

The HU derived from CBCT is a practical non-invasive tool for monitoring osseous healing, it is important to contextualize its measurement. Unlike conventional medical CT, CBCT-derived bone density values are generally higher and may not be directly compatible with absolute intensity values. Therefore, our interpretation, focusing on relative changes and trends over time rather than precise quantitative measurements on an absolute scale, remains the most robust approach. Despite these inherent differences, CBCT offers advantages of higher spatial resolution, lower radiation dose, and lower cost, making it a widely accepted and valuable clinical tool for 3D morphological analysis and density assessment in orthognathic surgery [9]. The high intraobserver reliability observed in our study (ICC > 0.90) further validates the consistency of the CBCT measurement protocol used here. The observed bone regeneration patterns confirm that healing proceeds reliably under the NM-Low Z plasty. However, due to the lack of a control group, the interpretation of these findings is limited to confirming the physiological healing response of this specific technique and should not be used to infer comparative efficacy against other BSSO methods.

At T2, the bone density in the cancellous and lingual cortical regions had recovered to levels comparable to those at T0. Thus, these regions demonstrated faster recovery compared to the buccal cortex. This is consistent with prior biological and clinical observations that cancellous bone, because of its rich vascularity and trabecular structure, possesses a high metabolic turnover rate, which inherently allows it to regenerate more rapidly than the dense cortical bone. Cancellous bone tends to heal more rapidly than cortical bone [10]. Furthermore, the lingual cortex, which was subjected to less periosteal stripping, showed earlier regeneration. This aligns with the findings of Moroi et al. (2016), who reported faster bone healing on the lingual side after BSSO owing to minimal periosteal elevation and reduced soft-tissue trauma [11]. However, according to the Lekholm and Zarb bone quality categories (1985) [12], and using the ranges adapted from Misch’s classification [13], the mean HU of the lingual cortical bone at T3 was consistent with D2 bone quality, which is the same as that at T0.

However, HU measurements obtained from CBCT may not represent absolute values on a numerical scale. Instead, they should be interpreted as relative indicators of bone density on an ordinal scale, reflecting differences between regions or time points rather than precise quantitative measurements.

Cancellous bone exhibited minimal change in HU values between T0 and T1. This could be explained by the inherent biological properties of cancellous bone. Owing to its rich vascularity and high metabolic turnover, cancellous bone has the capacity to regenerate rapidly after surgical trauma [10,14]. In fact, early bone healing in cancellous regions is typically characterized by the rapid formation of woven bone, which begins within a few days after osteotomy and progresses significantly during the first few weeks. This initial bone matrix serves as a scaffold for subsequent remodeling into lamellar bone, particularly under stable mechanical conditions and adequate vascularity [15]. Thus, the bone density at T1 may reflect the early stages of bone regeneration, resulting in HU values similar to those at T0. Additionally, the osteotomy gap is small in the cancellous area after NM-low Z plasty [4], and in early postoperative stages, it is often filled with provisional tissue such as blood clots, granulation tissue, and unmineralized matrix [16]. These components may exhibit radiodensity levels similar to those of cancellous bone. Therefore, the combination of early woven bone formation, a small gap, and the presence of tissue with similar radiodensity may contribute to the apparent similarity in HU values between T0 and T1.

Additionally, the pattern of gradual increase in HU over time observed in this study mirrors the bone healing trajectory described by Daif (2013) and Lettry et al. (2003) [17,18], in which a soft callus is evident within 1 month, followed by cortical consolidation and remodeling at 6–12 months. Our findings that HU reduces at 1 month (T1), followed by progressive increase toward baseline or normal values by 6–12 months (T2–T3), support this model. The return of buccal cortical density to preoperative levels at T3 highlights the ability of the NM-Low Z plasty technique to support full cortical remodeling.

The absence of postoperative complications such as infection, delayed union, or non-union highlights the clinical viability of this technique. This complication-free healing profile aligns with the biomechanical evidence presented by Dumrongwanich et al. (2022) [6], who demonstrated via finite-element analysis that the NM-Low Z plasty technique reduces stress at the fixation site compared to the conventional HE model, thereby supporting more favorable healing environments.

Radiographic confirmation of cortical bridging and HU recovery within 12 months supports the current clinical practice in which plate removal is considered safe at approximately 9–12 months postoperatively. This aligns with the findings of Little et al. (2015) and Sukegawa et al. (2018) [19,20], who reported high rates of plate removal within 1 year after BSSO with no adverse effects when adequate bone healing was confirmed. The timing of miniplate and screw removal after BSSO using the NM-Low Z plasty technique is a major clinical implication of this study. This study demonstrated that at T3, the bone density at the buccal, lingual, and cancellous regions of the osteotomy site returns to levels comparable to those at T0 or normal cortical values. These findings support the notion that the osteotomy gap is radiographically healed at this timepoint, and thus, removal of fixation hardware can be safely performed without compromising mandibular integrity. This is particularly relevant in clinical situations where patients report discomfort from hardware or request removal for personal reasons.

Surgical influence on HU increases at T3 in the lingual and cancellous regions. Interestingly, this study found that the mean HU at T3 in the lingual cortical and cancellous regions exceeded those at T0. This suggests not only successful bone regeneration but also possible increased mineralization or structural consolidation in these regions after surgical intervention. One possible explanation is the intraoperative bone manipulation during the NM-Low Z plasty. In many cases, cancellous bone is partially removed at the osteotomy interface to facilitate precise cortical contact and segment alignment, particularly in the buccolingual direction. This removal effectively brings the cortical plates—especially the buccal and lingual cortices—closer, potentially enhancing the environment for direct cortical healing and leading to denser bone formation postoperatively. In addition, on the lingual side, anatomical overlap between the proximal and distal segments often occurs during mandibular setback because of the vector of posterior displacement and curvature of the mandibular body. This cortical overlap may increase the surface area of the contact and reduce intersegmental gaps, both of which promote faster and denser cortical bridging [21]. Moreover, the close apposition of cortical bone may facilitate osteogenic activity and remodeling that ultimately yields higher HU values than those at baseline. These surgical and anatomical factors may contribute to the observation that both lingual and cancellous areas at T3 exhibit radiodensity levels exceeding those at T0. This finding provides crucial clinical confirmation that sufficient bone mineralization and consolidation have occurred by 10–12 months postoperatively, supporting this period as the safe and appropriate timing for elective removal of fixation hardware.

In this study, mandibular setback distances ranged from 1.84 mm to 25 mm. Despite the wide variation, no correlation was observed between the magnitude of mandibular setback and the degree of bone healing as assessed using HU values at T3. Notably, the patient who underwent the greatest setback (25 mm) exhibited the highest HU values across all measured regions—buccal cortex, cancellous bone, and lingual cortex—whereas another patient with a moderate setback of 22.58 mm exhibited the lowest overall bone density at T3. Conversely, patients with minimal setback distances also displayed a wide range of HU outcomes, suggesting that setback distance alone does not consistently predict bone regeneration quality. From regression analysis of the setback distance (Coef. −10.04), we found that the negative coefficient indicates a trend where greater setback is associated with slightly lower HU at T3. This aligns with the expectation that moving the segments further apart may slightly compromise vascularity or increase stress over time, although the effect is small and not statistically significant.

Regarding the relationship between the osteotomy gap width and bone healing at T3, although all gap measurements were within the clinically acceptable range for direct or gap healing (0.69–1.88 mm), a trend suggesting that larger gaps may be associated with slower bone regeneration, particularly in the cancellous and buccal cortical regions, was observed. For example, the patient with the largest osteotomy gap (1.88 mm) demonstrated the lowest Hounsfield unit (HU) values at T3, whereas patients with smaller gaps (0.78–0.81 mm) generally showed higher HU values across regions. Nonetheless, the relationship was not strictly linear. Some patients with gaps > 1.5 mm exhibited high bone density at T3, suggesting that other factors such as bone-segment alignment, cortical contact, vascular supply, and individual biological variability may modulate healing outcomes. Mean gap size (Coef. −807.33): This negative coefficient with a large magnitude highlights the strong inverse relationship trend: a larger mean gap size is associated with a dramatic drop in lingual bone density. This is directly relevant to the surgical technique. The negative coefficient suggests that when the intended cortical overlap or close apposition is not achieved (i.e., the mean gap is larger), the final healing outcome is severely compromised, emphasizing the paramount importance of meticulous technique in managing this gap. These findings are consistent with those of Claes, (1997) [22], who reported that gap sizes ≤2 mm can heal successfully through direct bone healing mechanisms if rigid fixation is achieved. Therefore, although minimizing the osteotomy gap is desirable, ensuring proper alignment and stability may be more critical in supporting optimal bone regeneration. These findings are consistent with those of previous studies, which emphasize that factors such as gap width, cortical bone contact, and biological healing capacity have a more significant influence on bone remodeling than the linear distance of mandibular movement [23]. In the context of the NM-Low Z plasty technique, where precise osteotomy design is achieved, optimal bone-segment contact and minimized trauma likely play more critical roles in promoting favorable healing outcomes than the setback distance. Therefore, although the extent of mandibular setback is an important surgical planning consideration, it does not appear to be an independent determinant of successful bone healing.

Suggesting new avenues for the future, other researchers should consider employing more complex regression models (e.g., non-linear regression, piece-wise regression) to investigate whether there is a critical threshold (cut-off point) for the osteotomy gap size. This is particularly relevant to determine if the healing process switches abruptly from direct bone healing to secondary bone healing mechanisms beyond a certain gap width. Our second suggestion is comparative efficacy and standardization. Future work should quantify the actual surgical advantage by comparing the achievable maximum setback distance using NM-Low Z plasty versus HE, alongside a concurrent measurement of bone density outcomes. Our third suggestion is to focus on biomechanical and biological predictors such as measurement of cortical overlap; the regression showed a high R^2^ (0.774) in the lingual cortex, suggesting that gap management is critical. Future studies should precisely quantify the area of cortical overlap (in mm^2^) using CBCT. This quantitative overlap area should be used as a specific independent variable in regression analysis to determine its precise predictive power on T3 HU values, separating its effect from the simple linear gap size measurement. Our next suggestion is to use preoperative bone quality as a predictor by investigating the relationship between initial bone density (HU at T0) in each segment and the subsequent change in density. This would help determine if the patient’s inherent bone quality is a significant prognostic factor for the success of bone regeneration. The last suggestion is the use of long-term follow-ups to examine stability. Future work should extend the follow-up period to 24 months or 5 years. This is essential to evaluate if the enhanced mineralization (HU exceeding T0) observed in the lingual and cancellous regions is maintained over time and whether this increase in bone density correlates with improved long-term skeletal stability and reduced relapse rates.

## 5. Conclusions

The findings in this study indicate that bone regeneration after BSSO using the NM-Low Z plasty technique proceeds in accordance with the expected physiological healing process. The extent of mandibular setback did not significantly influence the healing outcome, suggesting that optimal segmental contact and stable fixation may offset the potential impact of extensive mandibular movements. Conversely, the osteotomy gap size significantly affected bone regeneration, with larger gaps generally associated with delayed mineralization. However, this relationship was not strictly linear, implying that additional variables including cortical bone contact and vascular supply play contributory roles in determining the rate of osseous healing.

## Figures and Tables

**Table 1:** Patient demographics and surgical characteristics.

Case	Sex	Age (years)	Setback Amount (mm)	Osteotomy Gap Size (mm)		
				Buccal	Cancellous	Lingual
**1**	Male	24	25	1.17	1.07	1.35
**2**	Male	30	13.93	1.35	0.81	1.42
**3**	Male	23	4.51	1.34	1.70	0.83
**4**	Female	33	3.33	1.06	0.78	1.66
**5**	Female	26	22.58	0.9	1.88	0.93
**6**	Male	36	1.84	1.05	1.06	0.69
**Mean ± SD**	—	28.67 ± 5.20	11.87 ± 10.19	1.145 ± 0.18	1.22 ± 0.46	1.15 ± 0.38

SD, standard deviation.

**Table 2:** Intraobserver reliability test.

Measure Type	ICC Value	95% CI (Lower–Upper)	F-Value	*p*-Value
Average Measures	0.991	0.986–0.995	114.098	<0.001

ICC, intraclass correlation coefficient.

**Table 3:** Test of normal distribution by using Shapiro–Wilk test.

Test	Statistical Value	*p*-Value
Buccal cortex	0.934	0.120
Cancellous	0.951	0.291
Lingual cortex	0.941	0.169

**Table 4:** Pairwise comparisons at buccal cortex, cancellous bone, and lingual cortex.

Timepoints Compared	Buccal Cortex		Cancellous		Lingual Cortex	
	Mean Difference	*p*-Value *	Mean Difference	*p*-Value *	Mean Difference	*p*-Value *
T0/T1	1187.833 ^†^	0.006	222.122	0.578	705.845 ^†^	<0.001
T0/T2	953.833 ^†^	0.005	−52.272	1.000	193.850	1.000
T0/T3	406.217	0.321	−185.194	0.657	−151.267	1.000
T1/T2	−234.000	0.822	−274.394 ^†^	0.030	−511.995	0.080
T1/T3	−781.617 ^†^	0.023	−407.137 ^†^	0.006	−857.112 ^†^	0.002
T2/T3	−547.617 ^†^	0.020	−132.922	0.106	−345.117	0.088

* *p*-value of pairwise comparison (post hoc) between groups. ^†^ Mean difference <0.05 was considered statistically significant.

**Table 5:** Summary of multiple linear regression analysis.

Measured Region (T3)	R^2^	*p*-Value (F Test)	Setback *p*-Value	Mean Gap *p*-Value	Setback Coefficient	Mean Gap Coefficient
T3 Buccal	0.083	0.879	0.702	0.926	+2.20	+43.27
T3 Cancellous	0.02	0.97	0.834	0.866	+2.65	−175.85
T3 Lingual	0.774	0.107	0.178	0.187	−10.04	−807.33

R^2^ is Coefficient of Determination.

## Data Availability

The data supporting the findings in this study are available from the corresponding author upon reasonable request.
